# When Revascularization May Be Appropriate in Atherosclerotic Renal Artery Stenosis

**DOI:** 10.7759/cureus.64854

**Published:** 2024-07-18

**Authors:** Michael N Zarrella, Kolu Wynne, Phelese Smith, Shrimahitha Duraiyarasan, Mehmet A Elbey

**Affiliations:** 1 Internal Medicine, St Mary's Hospital, Waterbury, USA

**Keywords:** angiotensin-converting enzyme inhibitors (acei), chronic kidney disease, optimized medical therapy, antiplatelet therapy, angiotensin receptor blocker (arb), acute kidney injury, flash pulmonary edema, renal failure, syndromes of cardiac destabilization, atherosclerotic renal artery stenosis

## Abstract

Renal artery stenosis (RAS) is a condition that involves the narrowing of one or both renal arteries, most commonly caused by either atherosclerosis or fibroplasia. RAS can present in a multitude of clinical manifestations involving hypertension (HTN), heart failure, and renal failure. Current recommendations for treating patients with RAS involve strict medical therapy often without invasive therapies. However, in more complicated patients with RAS, recent clinical studies and guidelines have offered varying recommendations, which has presented challenges in managing these cases. This review aims to summarize current evidence to best evaluate which patients with RAS may benefit from renal artery revascularization as opposed to medical therapy alone.

## Introduction and background

In recent years, the clinical management of atherosclerotic renal artery stenosis (ARAS) has become increasingly complex. Notable randomized controlled trials, ASTRAL and CORAL, have demonstrated no significant difference between groups that were revascularized as compared to traditional medical therapy alone [1,2]; however, there has been newly developing evidence to support invasive therapy for certain patient groups. In 2018, the Journal of American College of Cardiology (JACC) released criteria for establishing patient populations that will or may benefit from revascularization. Those patients who show benefit from revascularization are those with advanced ARAS presenting with worsening complications secondary to disease progression, such as flash pulmonary edema or rapidly declining renal function [3]. While first-line management remains medicinal therapy, there is developing evidence to support revascularization in clinical practice.

## Review

Pathophysiology****

Renal artery stenosis (RAS) is defined as the narrowing of one or both renal arteries. The degree of narrowing to be deemed hemodynamically significant is defined as stenosis greater than 70-80% renal artery luminal obstruction or with transluminal gradients significant enough to produce physiologic effects [3,4]. Clinically significant or hemodynamically significant narrowing of the renal artery produces physiological effects such as hypertension (HTN), chronic kidney disease (CKD), and heart failure [5]. RAS is most commonly caused by diffuse atherosclerosis, typically affecting the proximal main renal artery [6]. RAS may also be caused by fibromuscular dysplasia (FMD), which is common in women less than 50 years of age and typically involves the mid-renal and distal-renal arteries and/or branches [6]. 

FMD is a non-inflammatory condition that predominantly affects the carotid and renal arteries, with a prevalence of 3-4% in affected renal arteries [4,7,8]. FMD typically occurs in young females between 30 and 50 years of age [6]. The majority of RAS secondary to FMD involve the middle portion of the vessel and can cause substantial stenosis that is greater than 80%; however, it rarely causes complete vessel occlusion or ischemic atrophy of the affected kidney [1].

Atherosclerotic renal artery disease (ARAS) is a primary disease characterized by endothelial injury and activation of inflammatory pathways resulting in the accumulation of lipids, fibrosis, and calcification of the vessel [9]. ARAS occurs in both large and medium arteries and typically presents with bilateral plaques. ARAS characteristically involves the proximal portion of the renal artery and is often associated with atherosclerosis of the abdominal aorta [10]. Men are more commonly affected than women with the prevalence being roughly 2:1 [1]. Common clinical presentations of ARAS include heart failure, HTN, flash pulmonary edema, acute kidney injury (AKI) with subsequent development of CKD, and encephalopathy. 

In patients with RAS, HTN is the root cause of secondary physiologic effects with the driving mechanism being the renin-angiotensin-aldosterone system (RAAS) [11]. Renin enters the circulation from the juxtaglomerular (JG) cells lining the afferent arterioles of the kidneys and plays a major role in the RAAS. Renin plays a physiologic role in converting angiotensinogen to angiotensin I [12]. Angiotensin I is then converted to angiotensin II by angiotensin-converting enzyme expressed in the pulmonary vasculature. This now-active molecule angiotensin II produces a multitude of effects to elevate blood pressure in the way of increasing peripheral vascular resistance and increased sodium and water reabsorption at the level of the kidney via the stimulation of aldosterone [5]. Angiotensin II can inhibit renin production at JG cells via a negative feedback mechanism [13]. Angiotensin II stays active for a very short duration of time with its half-life being less than 60 seconds [14]. Aldosterone further promotes sodium and water reabsorption and provides an additive effect on increasing blood pressure. Similarly, RAAS also stimulates the sympathetic nervous system, which contributes to systemic vasoconstriction and increased blood pressure [5]. The pathophysiology of RAS is significant as it is utilized in targeted medical therapies.

Clinical presentations of RAS

In a clinical setting, patients who present with RAS may have a wide array of symptomatology. Most commonly, ARAS presents as secondary HTN that is often resistant to antihypertensive medications [5]. 

HTN

ARAS is one of the leading causes of secondary HTN resulting in complications, such as flash pulmonary edema, or deterioration of the renal function [15,16]. Certain indicators (Class I) that may raise clinical suspicion for HTN secondary to RAS include new-onset HTN in a patient less than 30 years of age or greater than 55 years of age, accelerated or resistant HTN, declining renal function, development of azotemia after medication administration, atrophic kidney or size discrepancy greater than 1.5 centimeters between the two kidneys, and sudden onset pulmonary edema [17]. Other indicators include unexplained renal failure (Class IIa), multivessel coronary artery disease (Class IIb), and unexplained heart failure (Class IIb) [17]. Treatment-resistant HTN is defined as persistent HTN despite maximally tolerated doses of multiple antihypertensive medications and diuretics [4].

Cardiac Manifestations

ARAS is strongly associated with causing heart failure as prior evaluations have demonstrated up to 54% of patients with systolic heart failure had evidence of renal artery disease [4]. Resistant HTN can result in hospitalizations as manifested in patients presenting with recurrent heart failure, acute coronary syndrome, or pulmonary edema [4,18]. 

AKI and CKD

ARAS can present with unexplained rapidly progressive AKI in patients with HTN [4]. Worsening ARAS can eventually result in ischemic nephropathy and associated CKD [4]. Similarly, patients may have signs of hyperaldosteronism with low potassium, AKI, and HTN [19]. Patients with severe AKI may end up requiring rescue hemodialysis [20]. 

Encephalopathy

When the stenosis is severe enough to critically reduce renal blood flow, patients can develop hypertensive urgency, along with grade 3-4 Keith Wagener Barker (KWB) retinal changes, and hypertensive encephalopathy (which can present as stroke, convulsions, and unconsciousness), referred to as hypertensive vascular crisis [21,22].

Medical management

Clinical guidelines from the American College of Cardiology (ACC), American Heart Association (AHA), and European Society of Cardiology have shown substantial evidence of utilizing medical therapy as the standard of care for managing ARAS. Recommendations begin with establishing blood pressure goals <130/80 millimeters of mercury (mmHg). The principle of pharmacotherapy relies on the blockade of RAAS through the use of angiotensin-converting enzyme inhibitors (ACEi) and angiotensin receptor blockers (ARB), both of which reduce HTN and the progression of renal disease [4]. Both ACEi and ARB lines of therapy have shown mortality benefits in the reduction of myocardial infarction or stroke when compared to similar patients provided with other medical therapies [23]. Additional management includes eliminating risk factors for ARAS progression and prevention of cardiovascular or renal events. This is achieved through the use of lipid-lower agents, typically statins, and aspirin, which are advised for their preventative effects. If applicable, appropriate glycemic control for patients with diabetes should be emphasized. In the same regard, patients with a history of cigarette smoking should be counseled extensively on smoking cessation to prevent disease progression and reduce the risk of cardiovascular events [4].

Clinical suspicion of ARAS should be high when evaluating patients who have treatment-resistant HTN associated with CKD [5]. Diagnostic investigations of ARAS often rely on imaging studies to determine the degree or severity of RAS. Duplex ultrasonography is typically used as a first-line study as it can quickly provide information about renal arterial blood flow velocities to determine if a more detailed workup may be warranted. In addition, the measurement of the intrarenal resistance index by duplex ultrasonography can be helpful in evaluating the severity of stenosis [24,25]. Computed tomography angiography (CTA) imaging and magnetic resonance angiography (MRA) are also valuable in assessing ARAS. The gold standard is catheter angiography, which is typically reserved for more significant cases when revascularization is being considered an option in management [5].

Different interventional treatment options available for ARAS are medical therapy (antihypertensive medications, drugs to control atherosclerosis), percutaneous therapy (balloon angioplasty and stenting), surgical treatment (bypass surgery), or endarterectomy [26]. First-line management for RAS is medical therapy, revolving around the control of the RAAS through methods of either ARB or ACEi.

Clinical trials and current guidelines

Some of the most conclusive data has resulted in recent years regarding the management of patients with RAS. While FMD is typically managed medically with antihypertensive and antiplatelet agents, the management of ARAS is more expansive. Two of the most significant randomized control studies conducted were those of the ASTRAL and CORAL trials, both of which found no substantial mortality benefit to revascularization patients with atherosclerotic RAS [1,2]. 

ASTRAL Trial

A multicentered randomized control trial released in 2009 involving 806 participants with atherosclerotic renovascular disease was composed with the intent to evaluate if there is clinical benefit in revascularization. Patients were randomly divided into two groups: medical therapy alone or revascularization along with medical therapy. Parameters assessed included blood pressure, renal function, and renal and cardiovascular events and mortality. The conclusions drawn were that revascularization was not associated with any significant benefit to blood pressure, renal function, renal or cardiovascular events, or mortality [2].

CORAL Trial

A follow-up to the ASTRAL trial, the CORAL trial released in 2013 was a randomized control trial that aimed to evaluate medical therapy alone versus revascularization in addition to medical therapy. The study involved 947 participants randomly assigned to either treatment group with the intent of evaluating if renal artery stenting provided benefit in mortality, HTN, or CKD in ARAS. The evaluation found that there was no significant benefit in HTN, renal function, or mortality between medical therapy alone as compared to revascularization in addition to medical therapy [1]. 

JACC Appropriate Use Criteria for Peripheral Artery Intervention

In 2018, guidelines were released for managing patients with peripheral arterial disease from the collaborative effort of the ACC, AHA the Society for Cardiovascular Angiography and Interventions (SCAI), the Society of Interventional Radiology, and the Society for Vascular Medicine. Together, these organizations have established diagnostic criteria known as Appropriate Use Criteria (AUC), which was used to assess clinical scenarios in determining when revascularization may be appropriate. These parameters were ascertained from a variety of published guidelines and expert opinions within the field of peripheral vascular disease. The publication's goal was to help provide clinicians guidance in deciding what patients are most likely to benefit from interventional therapies, such as revascularization [3].

As it pertains to ARAS, the AUC bases recommendations off of patients with hemodynamically significant RAS defined as RAS with 70-99% stenosis or RAS with 50-69% stenosis associated with hemodynamic significance [3]. Through this, the AUC divides these patients into multiple categories, including cardiovascular, CKD, and HTN, in providing recommendations for revascularization.

Patient selection

Distinguishing which patients may be appropriate for stenting is entirely dependent on an individual case basis. Patients who are hemodynamically stable and have a known diagnosis of RAS should be managed medically; however, long-term medical management has varying outcomes. Mortality increases in patients with worsening baseline renal insufficiency, therefore highlighting the benefit of early ARAS diagnosis and management [27].

According to the AUC, scenarios in which revascularization is appropriate are for patients who present with acute decompensated heart failure with flash pulmonary edema and also for those who have bilateral RAS alongside worsening kidney function [3]. Similarly, scenarios in which revascularization may be appropriate include patients with cardiovascular findings defined as recurrent heart failure exacerbations or uncontrolled unstable angina despite optimized medical therapy. Additional recommendations related to patients with CKD who have unilateral RAS associated with worsening renal function [3]. A meta-analysis study showed that renal artery stent placement could help solitary functioning kidney patients stabilize or even improve their renal function with a benefit rate of 77% [28]. Finally, patients with HTN who remain uncontrolled despite optimized medical therapy warrant potential indication for revascularization [3].

On the contrary, the presence and severity of baseline kidney injury are associated with increased mortality in ARAS patients [27]. Therefore, in truly assessing mortality benefits in patients prior to revascularization, baseline renal function plays a confounding role and should be considered in patient selection for mortality benefit following stenting. ​​​​​​​Baseline renal function is particularly important as it pertains to considerations of stenting as the potential insult of intra-operative contrast-induced kidney injury [4].

**Figure 1:**
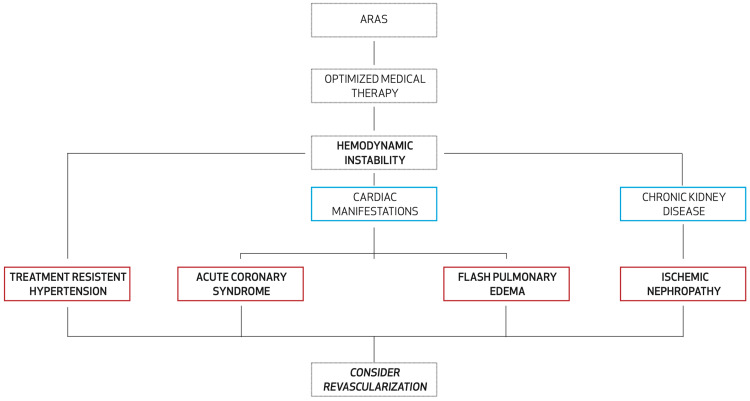
Management of ARAS and when to consider angioplasty *Hemodynamically significant atherosclerotic renal artery disease (ARAS) is defined as stenosis greater than 70% or less than 70%, but with transluminal gradients significant enough to produce physiologic effects. *Optimized medical therapy: angiotensin-converting enzyme inhibitor (ACEi) and angiotensin receptor blocker (ARB), lipid-lowering agent, antiplatelet agent, smoking cessation, and glycemic control.

Deciphering which patients may benefit from stenting is primarily based on whether stent placement will directly improve blood pressure and prevent the progression of renal impairment or recurrence of disease state, as demonstrated in Figure 1. In addition, patients who fall into these parameters have a decreased risk of developing both cardiovascular and renal events [3].

## Conclusions

Overall, advanced ARAS remains a challenging condition to manage for clinicians. Guideline-directed medical therapy (GDMT) continues to remain first-line and relevant for all cases. Renal artery interventions for revascularization have become more applicable to ARAS in patients who are critically ill or whose GDMT has failed. Evidence now demonstrates that while not first-line therapy, revascularization remains an option for patients with more complicated cases of ARAS, and as with any intervention, all recommendations should be considered on a case-by-case basis.

## References

[1] Cooper CJ, Murphy TP, Cutlip DE (2014). Stenting and medical therapy for atherosclerotic renal-artery stenosis. N Engl J Med.

[2] Wheatley K, Ives N, Gray R (2009). Revascularization versus medical therapy for renal-artery stenosis. N Engl J Med.

[3] Bailey SR, Beckman JA, Dao TD (2019). ACC/AHA/SCAI/SIR/SVM 2018 appropriate use criteria for peripheral artery intervention: a report of the American College of Cardiology Appropriate Use Criteria Task Force, American Heart Association, Society for Cardiovascular Angiography and Interventions, Society of Interventional Radiology, and Society for Vascular Medicine. J Am Coll Cardiol.

[4] Hicks CW, Clark TW, Cooper CJ (2022). Atherosclerotic renovascular disease: a KDIGO (Kidney Disease: Improving Global Outcomes) controversies conference. Am J Kidney Dis.

[5] Khan AR, Sheikh M, Kaw D, Cooper CJ, Khouri SJ (2014). Prevalence and factors associated with left ventricular remodeling in renal artery stenosis. J Am Soc Hypertens.

[6] Safian RD, Textor SC (2001). Renal-artery stenosis. N Engl J Med.

[7] Olin JW, Froehlich J, Gu X (2012). The United States Registry for Fibromuscular Dysplasia: results in the first 447 patients. Circulation.

[8] Sethi SS, Lau JF, Godbold J, Gustavson S, Olin JW (2014). The S curve: a novel morphological finding in the internal carotid artery in patients with fibromuscular dysplasia. Vasc Med.

[9] Jebari-Benslaiman S, Galicia-García U, Larrea-Sebal A (2022). Pathophysiology of atherosclerosis. Int J Mol Sci.

[10] Tafur-Soto JD, White CJ (2015). Renal artery stenosis. Cardiol Clin.

[11] Gomez J (2021). Renin angiotensin aldosterone system functions in renovascular hypertension.

[12] Martini AG, Danser AH (2017). Juxtaglomerular cell phenotypic plasticity. High Blood Press Cardiovasc Prev.

[13] Kurtz A, Wagner C (1999). Regulation of renin secretion by angiotensin II-AT1 receptors. J Am Soc Nephrol.

[14] van Kats JP, de Lannoy LM, Jan Danser AH, van Meegen JR, Verdouw PD, Schalekamp MA (1997). Angiotensin II type 1 (AT1) receptor-mediated accumulation of angiotensin II in tissues and its intracellular half-life in vivo. Hypertension.

[15] Vipparla N, Kichloo A, Albosta MS, Aljadah M, Wani F, Lone N (2020). Resistant hypertension secondary to severe renal artery stenosis with negative duplex ultrasound: a brief review of different diagnostic modalities. J Investig Med High Impact Case Rep.

[16] Prince M, Gupta A, Bob-Manuel T, Tafur J (2020). Renal revascularization in resistant hypertension. Prog Cardiovasc Dis.

[17] Colyer WR, Eltahawy E, Cooper CJ (2011). Renal artery stenosis: optimizing diagnosis and treatment. Prog Cardiovasc Dis.

[18] Prince M, Tafur JD, White CJ (2019). When and how should we revascularize patients with atherosclerotic renal artery stenosis?. JACC Cardiovasc Interv.

[19] Jegatheswaran J, Hadziomerovic A, Ruzicka M (2021). Acute severe renal artery stenosis presenting as acute kidney injury with severe hypertension and active urine sediment. Can J Cardiol.

[20] Abu-Amer N, Kukuy OL, Kunin M, Holtzman EJ, Rimon U, Dinour D, Beckerman P (2022). Treatment of severe renal artery stenosis with acute kidney injury requiring hemodialysis by percutaneous transluminal renal angioplasty and stent implantation. J Vasc Interv Radiol.

[21] Scarpelli P, Livi R, Caselli G, Gallo M, Teghini L, Becucci A, Rega L (1998). Hypertensive vascular crisis secondary to chronic total renal artery occlusion. J Nephrol.

[22] Antoniuk SA, Bruck I, Morais RL, Navolar FB, Meister E, Spessatto A (2000). Hypertensive encephalopathy associated to repetitive seizures: case report [Article in Portuguese]. Arq Neuropsiquiatr.

[23] Hackam DG, Duong-Hua ML, Mamdani M, Li P, Tobe SW, Spence JD, Garg AX (2008). Angiotensin inhibition in renovascular disease: a population-based cohort study. Am Heart J.

[24] Noory E, Rastan A, Beschorner U, Macharzina R, Zeller T (2016). Duplex derived intrarenal resistance index correlates with invasive pressure gradient measurements in detecting relevant unilateral renal artery stenosis. Vasa.

[25] de Leeuw PW, Postma CT, Spiering W, Kroon AA (2018). Atherosclerotic renal artery stenosis: should we intervene earlier?. Curr Hypertens Rep.

[26] Triantis G, Chalikias GK, Ioannidis E, Dagre A, Tziakas DN (2022). Renal artery revascularization is a controversial treatment strategy for renal artery stenosis: a case series and a brief review of the current literature. Hellenic J Cardiol.

[27] Bates MC, Campbell JE, Stone PA, Jaff MR, Broce M, Lavigne PS (2007). Factors affecting long-term survival following renal artery stenting. Catheter Cardiovasc Interv.

[28] Ma Z, Liu L, Zhang B, Chen W, Yang J, Li H (2016). Renal artery stent in solitary functioning kidneys: 77% of benefit: a systematic review with meta-analysis. Medicine (Baltimore).

